# Struma Ovarii during Pregnancy

**DOI:** 10.3390/diagnostics14111172

**Published:** 2024-06-01

**Authors:** Gabriela Dumachița-Șargu, Răzvan Socolov, Teodora Ana Balan, Dumitru Gafițanu, Mona Akad, Raluca Anca Balan

**Affiliations:** 1 Department of Morphofunctional Sciences I, “Grigore T. Popa” University of Medicine and Pharmacy, 700115 Iasi, Romania; sargu_gabriela@yahoo.com (G.D.-Ș.); raluca.balan@umfiasi.ro (R.A.B.); 2 “Elena Doamna” Clinical Hospital of Obstetrics and Gynecology, 700398 Iasi, Romania; socolovr@yahoo.com (R.S.); dgafit@yahoo.com (D.G.); 3 Department of Mother and Child Medicine, “Grigore T. Popa” University of Medicine and Pharmacy, 700115 Iasi, Romania; akad.mona@yahoo.com

**Keywords:** struma ovarii, pregnancy, thyroid carcinoma

## Abstract

Struma ovarii is a rare type of ovarian teratoma primarily composed of over 50% thyroid tissue. Its occurrence is reported in 2–5% of all ovarian teratomas, with approximately 0.5% to 10% showing malignant transformation. Managing it during pregnancy poses significant challenges as pregnancy can promote the growth of malignant struma ovarii due to elevated levels of ovarian and pregnancy-related hormones, including estrogen, progesterone, and human chorionic gonadotrophin (hCG). Most ovarian tumors, including struma ovarii, are detected during routine ultrasonography in the first and second trimesters, often as acute emergencies. Diagnosis during pregnancy is rare, with some cases incidentally discovered during cesarean section when inspecting the adnexa for ovarian cysts. This review explores the diagnostic, management, and therapeutic approaches to struma ovarii during pregnancy.

## 1. Introduction

First described in 1895 by Boettlin, who revealed thyroid follicular tissue in ovaries [1], struma ovarii represents a rare type of ovarian teratoma that predominantly (more than 50%) or exclusively contains thyroid tissue. This unique tumor typically occurs in individuals in their fifth and sixth decades of life [2]. They can be categorized as either mature or immature [3]. Mature variants, which make up approximately 20% of all ovarian teratomas, are composed exclusively of mature adult type tissues not native to the ovary, such as hair, skin, teeth, cartilage, bone, and thyroid tissue. Among mature ovarian teratomas, struma ovarii is the most common monodermal teratoma, representing 2–5% of ovarian teratomas and 1% of all ovarian tumors [4].

Although most struma ovarii are benign, they can present similar features with malignant tumors, such as distant metastases, a phenomenon considered as peritoneal strumosis [5].

The thyroid tissue found in ovarian teratomas can exhibit histological features similar to normal, hyperplastic/adenomatous, or carcinomatous thyroid tissue, either alone or in combination. Moreover, struma ovarii is associated with other ovarian tumors, such as carcinoid, mature cystic teratoma, mucinous cystadenoma, and Brenner tumor [6].

Even though the term “thyroid carcinoma originating in struma ovarii” is currently preferred instead of “malignant struma ovarii”, the latter is still commonly used in order to emphasize the malignization of benign thyroid tissue [7].

Determining the true incidence of thyroid carcinoma originating in struma ovarii remains challenging, being estimated to represent between 0.5% to 10% of all cases diagnosed with struma ovarii [7]. In this regard, understanding the diagnostic and clinical management of this condition, especially when it occurs during pregnancy, is critical.

For this review, a literature search was conducted using the main scientific databases (Web of Science, Scopus, Science Direct, Google Scholar, Cochrane Database for Systematic Reviews, PubMed/Medline) and acquisition was based on a database search using the following keywords: ‘struma ovarii’, ‘pregnancy’, ‘thyroid carcinoma’ in all the relevant combinations. In the next step of the literature selection, all the relevant full content English written literature in a time frame of 40 years was assessed, using as inclusion criteria only pregnancy-related struma ovarii, excluding pregnancy after treated struma ovary. Three retrospective studies, each one with only one diagnosed case of pregnancy-related struma ovarii, and 23 case reports were found.

Our personal experience in this pathology is reflected by three diagnosed and treated cases of struma ovarii, within a 15-year period, only one being associated with an early stage of pregnancy (first trimester).

This review aims to provide a summarized overview of the current state of knowledge regarding the diagnostic, management, and therapeutic approaches to struma ovarii during pregnancy, considering that only 26 publications have presented this topic, due to its rarity [4,5,8,9,10,11,12,13,14,15,16,17,18,19,20,21,22,23,24,25,26,27,28,29].

To the best of our knowledge, there is no published updated review related to di-agnostic and therapeutic management of struma ovarii during pregnancy.

## 2. Diagnostic and Clinical Management of Struma Ovarii during Pregnancy

Struma ovarii is rarely presented before the fifth and sixth decade of life, with only a few cases reported during pregnancy [4,5,8,9,10,11,12,13,14,15,16,17,18,19,20,21,22,23,24,25,26,27,28,29]. Adnexal masses occur with a frequency of approximately 0.2–2% during pregnancy, with dermoid cysts being the most common type, accounting for the majority of cases. It is worth noting that approximately 94% of these tumors are unilateral and seem to involve the left ovary more often than the right ovary [30]. Within this context, struma ovarii is a rare entity that requires thorough diagnosis and management, especially when it occurs during pregnancy. Although cases are rare, ovarian tumor masses must be monitored during pregnancy, especially when accompanied by clinical and paraclinical signs of hyperthyroidism, in order to diagnose the possible association with struma ovarii and to detect malignant transformation.

Hyperthyroidism accompanies 5–8% of all struma ovarii. Malignancy is uncommon, thyroid cancer representing less than 5% of all cases. A percentage of 5–23% distant metastases are reported in patients with thyroid carcinoma arising from struma ovarii [31].

When managing struma ovarii during pregnancy, clinicians must consider the well-being of both mother and fetus. This adds complexity to the clinical decision-making process. Factors such as the stage of pregnancy and the presence of symptoms play a crucial role in determining the appropriate treatment approach. 

The preoperative diagnosis of struma ovarii can be challenging. However, hyperthyroidism can be detected by measuring serum levels of thyroxine and thyroid-stimulating hormone. In most cases, post-operative histopathological assessment represents the tumor confirmation diagnosis.

While most patients do not experience symptoms, screening tests such as ultrasonography (US) often reveal pelvic masses. Patients may present with acute pelvic pain that may increase the risk for miscarriage or premature birth. Additionally, ascites, pleural effusion, and hyperthyroidism are occasional diagnoses in patients [32]. In some cases, the diagnosis is delayed until symptoms of ovarian torsion manifest [14].

Regular transvaginal ultrasound represents the most frequently used imaging ex-amination for ovarian masses during pregnancy. Ultrasound examination is a cost-effective method used during pregnancy to detect, diagnose, and monitor ovarian masses that may be associated with pregnancy. ISUOG (The International Society of Ultrasound in Obstetrics and Gynecology) recommends investigating the uterine adnexa starting from the first trimester of pregnancy to ensure early detection of any issues [33]. Recently, the screening rate of ovarian tumors associated with pregnancy has increased due to the potential complications that can worsen the prognosis of pregnancy, despite the low frequency of occurrence [34]. A systematic review by Gaughran et al. emphasizes the effectiveness of ultrasound in assessing adnexal masses during pregnancy, but due to insufficient evidence, it remains unclear if ultrasound is as reliable in pregnant patients as in pregnant ones [35].

**Figure 1:**
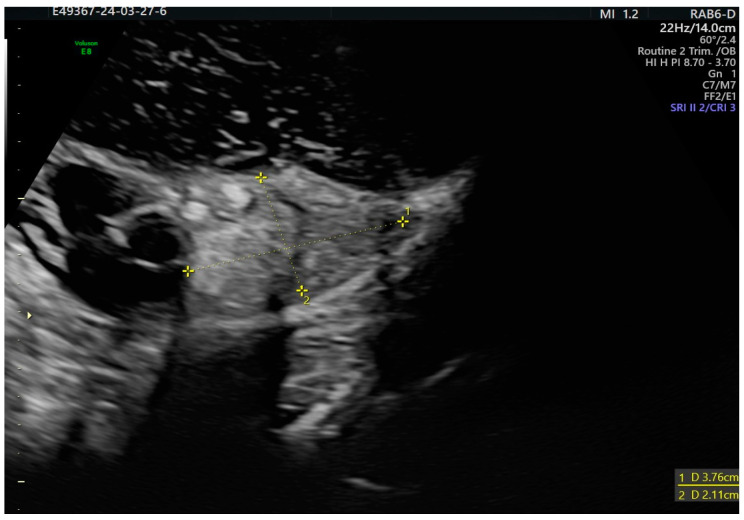
Struma ovarii in pregnancy appearing as a non specific multilocular solid tumor with two areas of “struma pearls”.

The specific feature of the struma ovarii is a well-vascularized solid tissue with smooth margin that appears vascularized in the Doppler study, often called a “struma pearl”, aspects also seen in our diagnosed case of struma ovarii in the context of pregnancy (Figure 1) [36,37].

The IOTA (International Ovarian Tumor Analysis) study group proposes different systems to quantify ovarian tumors, including IOTA simple rules, IOTA logistic regression models, and IOTA assessment of different neoplasias in the adnexa model (ADNEX) [38].

In this framework, benign ovarian tumors with thyroid-like characteristics are sonographically classified into two categories based on their anatomical features. Pure struma ovarii tumors typically appear as solid with some cystic components. They often have multiple compartments, some of which contain “struma pearls”. On the other hand, impure struma ovarii tumors have a mix of dermoid cyst and struma components. While they can be detected using ultrasound, their struma-like characteristics are less apparent. Generally, they appear as cystic or polycystic tumors with a solid component or “struma pearls” [36]. 

When applying the ovarian score system to pregnant women, it is important to note that ascites becomes more difficult to detect with ultrasound as gestational weeks increase. 

Additionally, the diagnosis of serum level markers during pregnancy remains controversial. The CA 125 level peaks in the first trimester and steadily decreases thereafter. However, slight elevations in CA 125 levels during pregnancy are not associated with malignancy. The prognostic value of CA 125 and HE4, another commonly used tumor marker, alone or in combination with ROMA (Ovarian Malignancy Risk Algorithm), is unknown during pregnancy [35]. 

Most ovarian tumors detected in the first trimester tend to regress by late pregnancy. However, clinicians should consider the possibility of malignancy for ovarian tumors to assure a proper therapeutic management [26].

MRI (magnetic resonance imaging) can be used in struma ovarii cases to reveal multilocular cysts with solid components of varying signal density. This imaging technique can enhance the detection rate of malignancy. However, the use of gadolinium, which is teratogenic, is limited during pregnancy. Furthermore, fetal movements can reduce image quality. Apart from initial extraovarian spread, factors indicating an increased risk of aggressive behavior in these tumors include adhesions to adjacent organs, significant ascites (1 L or more), strumal size larger than 5 cm, and the presence of more than 50% proliferating thyroid tissue within the teratoma [10]. 

When it comes to managing the metastasis and recurrence of struma ovarii, a comprehensive evaluation and close surveillance are crucial. A combination of serum tumor biomarkers, ultrasonography, and imaging scans such as computerized tomography (CT) or magnetic resonance imaging (MRI) are typically employed. It is important to note that tumor markers may not be accurate indicators of malignancy during pregnancy [15].

## 3. Genetic and Pathological Aspects of Struma Ovarii

Several genic mutations and rearrangements in thyroid carcinoma variants developed in struma ovarii have been revealed, such as RET/PTC and PAX8-PPARG rearrangements and BRAF and KRAS mutations [6]. 

A recent study on 13 benign and 40 malignant struma ovarii demonstrated, through molecular sequencing, BRAF mutations as highly suggestive for the malignant process. In this context, the p.G469A BRAF mutation was more commonly encountered in struma ovarii than in thyroid neoplasia, whereas the p.V600E variant was more frequent. Moreover, alterations of TERT promoter may correspond to a more aggressive character of the tumor [39].

**Figure 2:**
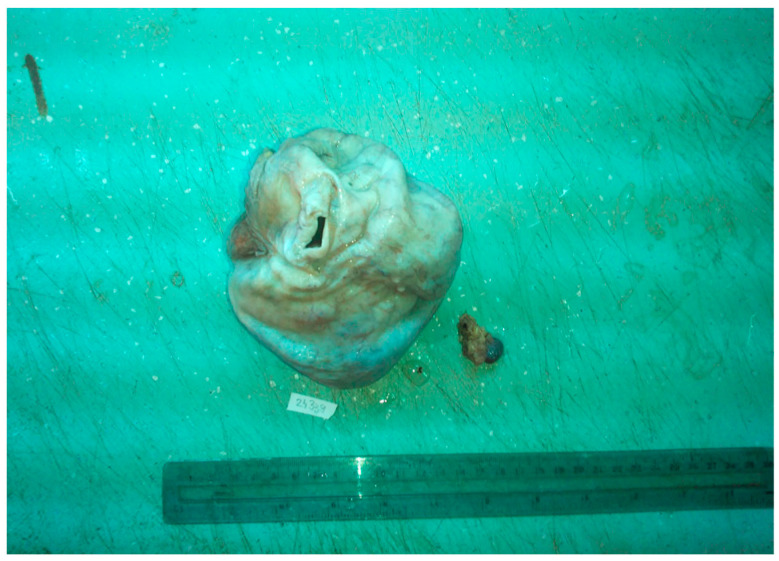
Solid tumor, with white surface.

**Figure 3:**
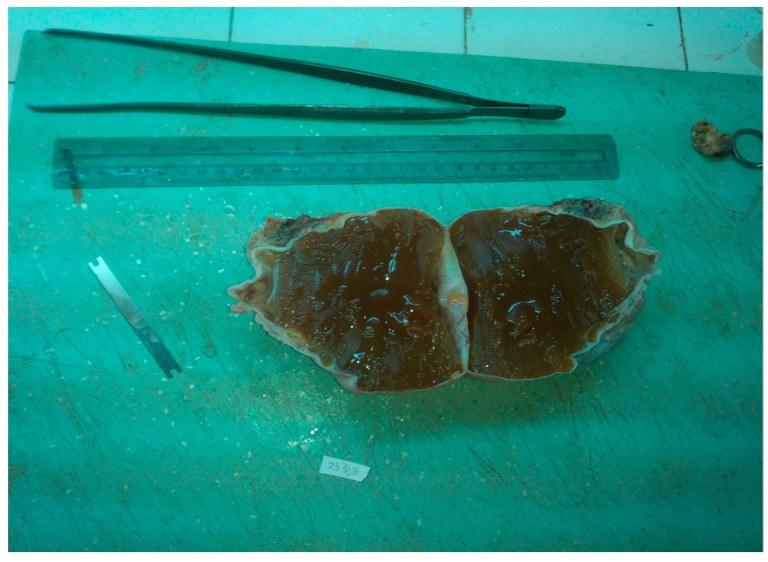
Struma ovarii, gelatinous, red–brown on cut surface.

Macroscopically, our pregnancy-related case of struma ovarii was unilateral and solid, measuring 10.5 cm in greater dimension, with white and smooth surface and a gelatinous, red–brown aspect on the cut section (Figure 2 and Figure 3), corresponding to the literature data where struma ovarii is described as mainly unilateral, with less than 10 cm in greater dimension, presenting a solid or sometimes cystic red–brown cut-surface. A dermoid cyst can frequently accompany the tumor [6].

Another intriguing case involves the coexistence of a round ligament dermoid cyst and struma ovarii during pregnancy. The development of these tumors in unusual lo-cations may be attributed to migration issues during the formation of germ cells. This case highlights the importance of thoroughly assessing the adnexa during cesarean section and removing any suspicious masses, regardless of size. Teratomas should also be considered in the differential diagnosis of pelvic masses, even in uncommon locations like the round ligament [25]. 

**Figure 4:**
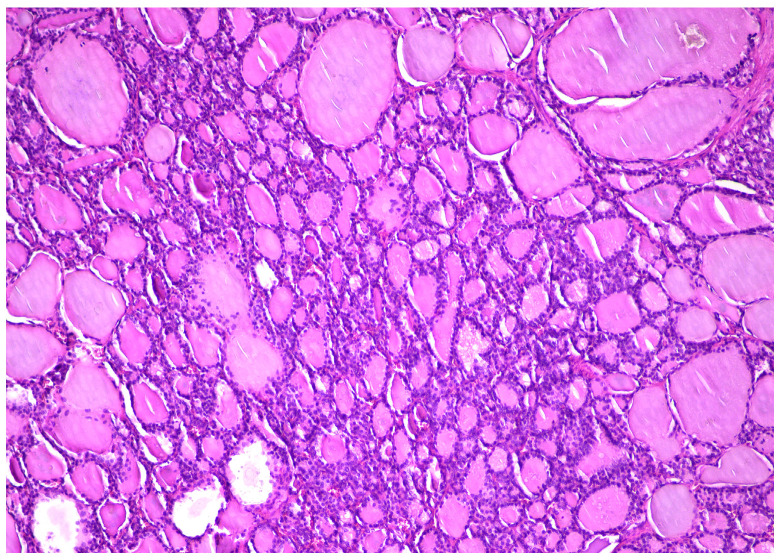
Different-sized thyroid follicles within struma ovarii, HE × 10.

**Figure 5:**
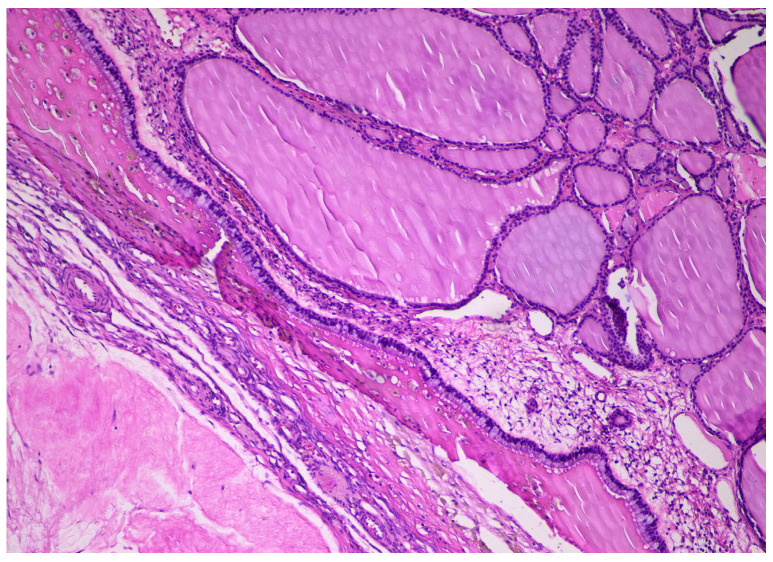
Different-sized thyroid follicles within struma ovarii, predominantly cystic, residual corpus albicans, HE × 10.

Histopathologically, our case presented specific morphological aspects, represented by normal thyroid tissue, with different-sized follicles filled with colloid and lined by cuboidal to flat cells, with scant cytoplasm and round small nuclei (Figure 4). Several patterns were described, including trabecular, solid, pseudotubular, pseudopapillary, and predominantly cystic (Figure 5), which sometimes can mimic a serous cystadenoma. As described in the literature, the stroma was usually scant and collagenous or oedematous, with peripherical luteinization [6].

Besides the two categories of benign ovarian tumors with thyroid-like characteristics, pure struma ovarii and impure struma ovarii, Savelli et al. describes, according to clinical ultrasound examination, a third category, struma ovarii with malignant focus, which appears as a solid mass with cystic spaces and irregular echogenic core [36].

Malignant struma ovarii exhibits the histopathological features of papillary thyroid carcinoma, presenting overlapped “clear” or “ground glass” nuclei, nuclear grooves, and papillary structures. Similar to the thyroid counterpart, the ovarian thyroid follicular carcinoma diagnosis is based on capsule or blood vessels invasion, neighboring organs infiltration, and distant metastasis. In the case of follicular malignant struma ovarii, the diagnostic criteria are difficult to apply. Adjacent ovarian tissue infiltration, lympho-vascular invasion, or metastasis represent morphological criteria for malignancy. Moreover, it is considered that Ki67 immunohistochemical assessment can provide aggressive-related features of the ovarian tumor [7].

## 4. Struma Ovarii Hyperthyroidism during Pregnancy

Struma ovarii causing hyperthyroidism is an uncommon clinical occurrence. The diagnosis is often suspected in patients presenting with clinical hyperthyroidism, negative scintiscans of the neck, and no history suggestive of thyroiditis, iodine contamination, or iatrogenic thyroid hormone consumption [40].

Unmonitored hyperthyroidism can be a serious risk factor for thyroid storm development during pregnancy [4,22]. Similar to the general population, pregnancy-related thyroid storm can be triggered by acute stressors such as surgery, infection, trauma, burns, hypoglycemia/diabetic ketoacidosis, and various drugs/toxins. Additionally, pregnancy-associated conditions like anemia, pre-eclampsia, placenta previa, induction of labor/C-section, and chorioamnionitis can also precipitate thyroid storm.

During pregnancy, the measurement of free or total triiodothyronine (T3), free or total thyroxine (T4), and thyroid-stimulating hormone (TSH) varies, according to the assay and trimester, the values being different from those in the general population. When the etiology is uncertain, it is advisable to assess hyperthyroidism by measuring TSH receptor antibodies (TRAb) using third-generation assays like thyrotropin-binding inhibitory immunoglobulin (TBII) or thyroid-stimulating immunoglobulin (TSI). TRAb are positive in patients with Graves’ disease, certifying its diagnosis [41,42,43].

## 5. Peritoneal Strumosis during Pregnancy

In peritoneal strumosis, struma ovarii may manifest as distant metastases, making them analogous to malignant tumors. Li et al. presented a case of a pregnancy-associated struma ovarii complicated with peritoneal strumosis, which has never been reported during pregnancy. In addition, pregnancy has been speculated to be a favorable environment for the progression of struma ovarii despite its rarity, based on the similarity between human chorionic gonadotropin and thyroid stimulating hormone [5].

The classification of struma ovarii with extraovarian dissemination remains a topic of debate among experts. Most authors consider that its malignancy is related to a highly differentiated follicular carcinoma (HDFCO) which originates from benign struma ovarii. HDFCO is composed of histologically benign thyroid follicles spreading beyond the ovary [44]. Thus, the metastatic potential gives this neoplasm a biologically malignant character. Moreover, the literature data suggest that peritoneal strumosis, which shares indistinguishable histological aspects with peritoneal and ovarian lesions, may represent a special type of HDFCO. However, further research is needed to fully understand this relationship [45].

## 6. Thyroid Carcinoma Originating in Pregnancy-Related Struma Ovarii (Pregnancy-Related Malignant Struma Ovarii)

Malignant degeneration of struma ovarii, a rare condition, can have significant implications for pregnant women. One article explores the various aspects of this condition and highlights the considerations that need to be taken into account when managing it during pregnancy [10].

The most frequent types of carcinomas originating from struma ovarii are papillary thyroid carcinoma (PTC) and follicular thyroid carcinoma (FTC) [6]. Follicular carcinoma usually metastasizes to the lungs, liver, and central nervous system, while papillary carcinoma commonly involves the abdominal cavity and lymph nodes. Although rare, anaplastic thyroid carcinoma and medullary thyroid carcinoma have also been reported in single case studies [8].

It is considered that pregnancy can stimulate the differentiated thyroid carcinogenesis. However, there are limited data to prove the development of thyroid carcinoma from struma ovarii during pregnancy. This may be due to the high levels of estrogen and hCG (human chorionic gonadotropin) during pregnancy, which can bind to receptors in malignant cells and stimulate TSH receptors [10]. Moreover, hCG stimulates TSH receptors, which are involved in ovarian function regulation, promoting carcinogenesis during pregnancy [10]. In one case of malignant struma ovarii it was revealed that serum thyroglobulin levels are higher during the first semester of pregnancy, decreasing in late pregnancy as well as in postpartum. These aspects are concordant with the hypothesis that pregnancy stimulates the malignant growth of ovarian thyroid tissue [10]. Nevertheless, data on the evolution of malignant struma ovarii during pregnancy remain scarce.

While the probability of thyroid cancer is slightly higher in pregnant women, there is no evidence suggesting that delaying the diagnosis of thyroid cancer until the post-partum period would raise the risk of malignancy. Thyroid cancer risk is temporarily influenced by pregnancy and lactation, metabolic, hormonal, or other specific factors [46,47]. There have only been seven case studies reported with malignant struma ovarii during pregnancy [8,10,11,15,24,48,49]. One explanation could be the estrogenic stimulation of benign and malignant thyroid cells [22]. In this regard, the diagnosis of malignant struma ovarii in a pregnant woman can be related to the stimulatory effect of pregnancy on the malignant thyroid tissue, and thus, in this context, the increased size of the tumor can be detected clinically [8,10,11,15,24,48,49].

There is no particular clinical management or standardized guidelines for thyroid carcinoma originating in struma ovarii. However, recent advancements in understanding the molecular mutations associated with these tumors have shed light on potential im-plications for identification and treatment.

To effectively diagnose and assess the metastasis and recurrence of struma ovarii, a multidisciplinary approach is necessary. Clinical manifestations, laboratory examinations, imaging information, and histopathology should all be considered. Although diagnosing ovarian masses during pregnancy can be challenging, any persistent mass should be evaluated to rule out the risk of malignancy.

The absence of correlation between morphology and outcome makes the management of proliferative and histologically malignant struma ovarii really challenging. This unpredictability makes it essential to closely monitor these tumors and adapt treatment plans accordingly.

## 7. Treatment of Pregnancy-Associated Struma Ovarii 

### 7.1. Treatment Options for Struma Ovarii during Pregnancy

In the realm of managing a rare condition like struma ovarii, there is currently no consensus on how to proceed after the initial surgical diagnosis. The limited number of cases and the absence of reliable prognostic factors contribute to the lack of clear guidelines for treatment. However, it is essential for all patients to undergo indefinite follow-up based on pathologic and imaging parameters, as well as individual characteristics. 

When a struma ovarii is diagnosed, two surgical techniques are taken into consideration: laparoscopic surgery or laparotomy and enucleation or oophorectomy. Enucleation is safer when the patient wants to preserve her fertility or is pregnant, but this technique increases the risk of intraoperative tumor rupture. Therefore, if the risk of detecting malignancy is eliminated, laparoscopic oophorectomy is indicated [50]. However, salpingo-oophorectomy or oophorectomy is often considered a better approach than laparoscopic surgery, because of the much lower risk of cyst rupture [50].

Li et al. reported a case of struma ovarii and peritoneal strumosis during pregnancy of a patient who had previously undergone ovarian cystectomy. The ovarian tumor, which was antepartum diagnosed, reappeared during next pregnancy, the disseminated masses being detected during the cesarean section. The tumor recurrence may be correlated with the conservative surgery performed for fertility preservation. Despite complete removal of the tumors, microscopic lesions cannot be ruled out. Although a rare occurrence, it has been speculated that pregnancy creates a favorable environment for struma ovarii progression [5].

An important aspect of pregnancy-related malignant struma ovarii is the patient’s desire for fertility. In thyroid cancer, estrogen, which stimulates the benign and malignant thyroid cells, may impact adhesion, migration, angiogenesis, and invasion. It is also known that estrogen and progesterone receptors are present in papillary thyroid cancer cells [51,52]. Furthermore, TSH receptors are stimulated by human chorionic gonadotropin (hCG), potentially promoting carcinogenesis during pregnancy [53]. Therefore, as it is possible for tumor size to increase during pregnancy, patients should be counseled regarding the potential increased risk.

Because of the rarity of these tumors and the absence of definitive prognostic factors, the therapeutic management should be personalized, according to clinical and pathological parameters.

### 7.2. Surgical and Prognostic Factors in Pregnancy-Related Struma Ovarii

When it comes to treating struma ovarii during pregnancy, the approach depends on factors such as gestational age, tumor size, and ultrasound findings. Surgery in the first trimester carries a higher risk of spontaneous abortion, so it should only be performed as an acute emergency or when there is a suspicion of malignancy. The potential adverse outcomes associated with surgery during pregnancy underscore the need for further research to determine the reliability of ultrasound in assessing adnexal masses. 

There are two surgical options for resecting ovarian tumors: laparotomy or laparoscopic surgery, and oophorectomy or enucleation. Laparoscopic surgery is less invasive, while enucleation offers the possibility of preserving the normal ovary and increasing the chance of pregnancy. However, enucleation presents challenges in avoiding tumor rupture. Moreover, enucleation may be considered when pregnancy preservation is the patient’s priority [54]. 

**Figure 6:**
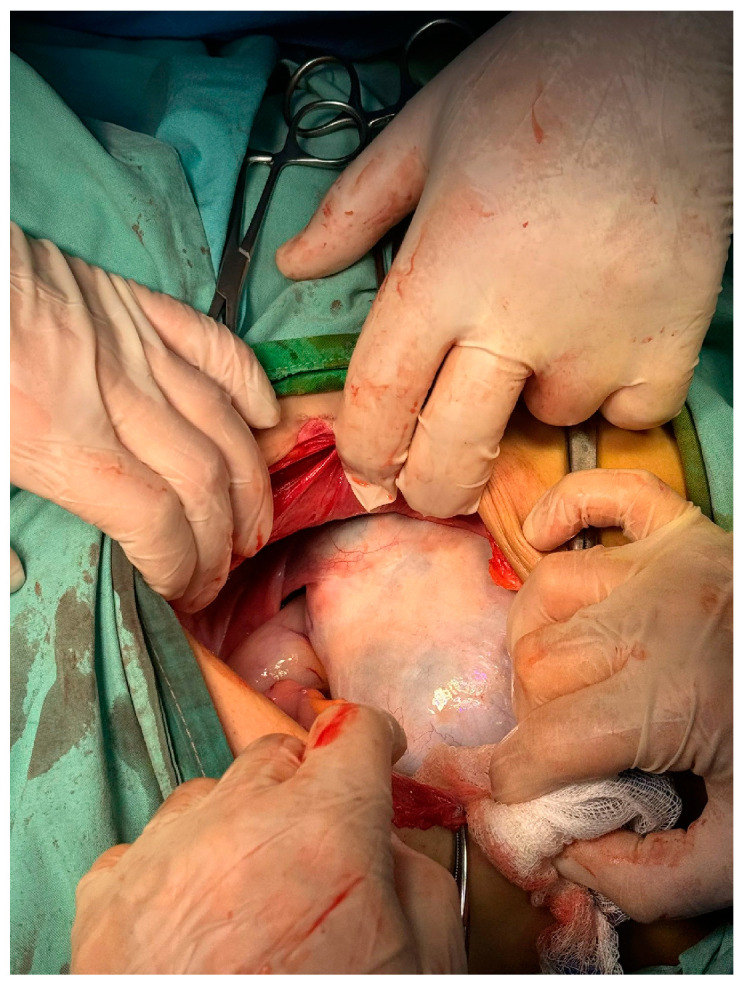
Intraoperatory view. Struma ovarii.

In our case we preferred laparotomy with oophorectomy as a surgical approach due to the large size of the tumor and the early stage of pregnancy (Figure 6).

### 7.3. Therapeutic Strategies in Pregnancy-Related Malignant Struma Ovarii

Integrating all available clinical, histopathological, laboratory, and diagnostic information is essential in order to make informed decisions about the management of advanced and metastatic thyroid cancer [23,55].

There is no standardized perspective for the diagnosis and management of malignant transformation of struma ovarii, especially in the context of pregnancy [48]. The surgical approach differs according to the patient’s age, the desire for fertility preservation, and sometimes the gestational context, varying between conservative surgery and total hysterectomy with bilateral adnexectomy, followed by chemotherapy and radiation. When a thyroid carcinoma developed from struma ovarii is diagnosed, thyroidectomy followed by adjuvant therapy with radioactive iodine is proposed [10,48,49].

For young patients diagnosed with malignant struma ovarii who wish to preserve their fertility, a conservative surgical approach is recommended. This typically involves unilateral salpingo-oophorectomy or ovarian cystectomy, guided by preoperative imaging findings. To ensure comprehensive treatment, the combination of laparoscopic tumor removal with thyroidectomy, followed by radiotherapy with I-131, should be considered. It is crucial to discuss this option thoroughly with the patient until more data about this rare condition become available [10,48,49].

Some articles suggest that in cases of thyroid carcinoma originating from struma ovarii, complete tumor removal, total thyroidectomy, and radioiodine therapy (I-131) should be performed. The measurement of serum thyroglobulin as a tumor marker for follow-up remains a controversial topic [56,57]. 

In the case of malignant struma ovarii with larger dimensions (more than 1 cm), Iodine-131 ablation therapy sometimes combined with total thyroidectomy has proven to be efficient. The advantage of thyroidectomy resides in histopathological confirmation of normal thyroid histology, excluding a primary thyroid carcinoma metastatic to the ovary. Secondly, after removing the normal thyroid tissue, radioactive iodine is afterwards taken into metastatic lesions. In this regard, the postablation total body scan is used to demonstrate the surgical excision status or to show the metastatic disease. Moreover, this principle permits the treatment of recurrent tumor [30].

It was shown that prognostic indicators for malignant struma ovarii include a tumor diameter greater than 16 cm, ascites, and adhesion to neighboring pelvic structures [2].

In one case report with metastatic struma ovarii during pregnancy, after undergoing a right ovarian cystectomy and incidental appendicectomy, the histopathological examination revealed follicular carcinoma within a teratoma, characterized by infiltrative growth and lymphovascular invasion. Post-delivery, abdominal computer tomography (CT) revealed liver metastasis and seeding nodules in the pelvic cavity. Surgical intervention successfully removed the metastatic lesions, followed by radioactive iodine therapy [38]. This case emphasizes the importance of prompt diagnosis and tailored treatment strategies, allowing for successful fertility preservation and subsequent management of metastatic disease.

### 7.4. Therapeutic Strategies for Fertility Preservation

Sadath et al. showed the first case of fertility preservation in a case of malignant struma ovarii achieved by controlled ovarian stimulation and embryo freezing. The patient initially underwent bilateral ovarian cystectomy, and upon discovering the malignant nature of the tumors, definitive treatment was planned, taking into consideration fertility preservation. Embryo freezing was achieved through controlled ovarian stimulation, resulting in the successful vitrification of two high-quality embryos. Following subsequent surgical interventions, including uterus-conserving surgery with bilateral oophorectomy, peritonectomy, omentectomy, appendicectomy and total thyroidectomy, the patient underwent embryo transfer and successfully delivered a healthy baby [58]. This article showcases the potential of assisted reproductive technologies in preserving fertility in patients with malignant struma ovarii.

Despite the outstanding advancements in fertility preservation for malignant struma ovarii, the challenges in the management of this pathology remain present. The lack of literature on fertility preservation before radical surgery underscores the need for further research in this area. Moreover, the prediction of ovarian response to ovarian stimulation or embryo quality can be challenging if the patients have a history of previous cystec-tomies or endometriosis [59]. Future studies should focus on refining protocols for fertility preservation and optimizing outcomes for patients with malignant struma ovarii.

The literature data revealed a notable case, when a patient diagnosed with malignant struma ovarii opted for conservative surgery to protect her fertility. The malignant struma ovarii with vascular invasion was confirmed by histopathological diagnosis, and peritoneal washing was positive for malignancy. Following a total thyroidectomy, radioactive iodine treatment, and daily thyroxine therapy, the patient experienced a spontaneous conception within months. She went on to have a successful delivery, defying the odds and proving that conservative surgery can lead to pregnancy even in the presence of malignant struma ovarii [60].

## 8. Discussion

Struma ovarii is a rare variant of ovarian teratoma which is characterized by the predominance of thyroid tissue as the main histological element.

Our review outlines the current state of knowledge regarding the diagnostic, management, and therapeutic approaches to struma ovarii during pregnancy, considering that only 26 publications have presented this topic, due to its rarity (Table 1) [4,5,8,9,10,11,12,13,14,15,16,17,18,19,20,21,22,23,24,25,26,27,28,29].

As long as the diagnostic and therapeutic approach in pregnancy-associated struma ovarii partially overlaps with the management of struma ovarii outside pregnancy, the review also refers to cases of ovarian struma not associated with pregnancy. 

In any case, the histopathological examination represents the diagnostic gold standard which decides the benign or malignant nature of the tumor, with an impact on subsequent therapeutic management. In this sense, the upcoming therapeutic approach will be different in the case of carcinoma, and, in addition, if struma ovarii is associated with pregnancy, the treatment strategy will consider the impact on both the mother and the fetus.

Ovarian tumors are not unusual in pregnancy, with most being detected in the first and second trimesters during routine ultrasonography or during cesarean section at the inspection of the adnexa. In some cases, struma ovarii was detected when the patient came for an obstetric condition: pain or vaginal bleeding as an acute emergency. When the struma ovarii is functional, it may give rise to hyperthyroidism with excessive vomiting in early pregnancy. 

Ultrasonography is predominantly used during pregnancy due to its relative safety and effectiveness in evaluating adnexal masses at that time. Advancements in technology have allowed for numerous studies to be conducted on scoring systems for evaluating mass malignancy using ultrasound characteristics in nonpregnant women. However, the application of a scoring system during prenatal care has yet to be established [36,37].

Performing MRI scans during pregnancy requires specific protocols and expertise from practitioners. Unfortunately, this can lead to unnecessary radical surgeries for patients with benign struma ovarii. To preserve fertility, laparoscopic surgery is recommended, but long-term postoperative follow-ups and investigations are necessary [10].

Differentiating between this neoplasm and malignant tumors can be challenging due to their morphological similarities and the rarity of struma ovarii. In the absence of thyrotoxicosis, the diagnosis is often delayed until the patient experiences symptoms such as ovarian torsion, ascites, or hemorrhage.

To rule out metastasis to the ovary from primary thyroid cancer, clinical thyroid examination and ultrasonography are essential, as the percentage of patients with co-existing primary thyroid carcinoma is unknown.

The goal of the surgical treatment, in a case of struma ovarii associated with pregnancy, is to be performed closer to the gestational age when baby’s lungs have matured as well as close to the expected date of birth. This can be achieved by preventing premature uterine contractions, which may be caused by the presence of an ovarian tumor. To extract the fetus and perform an adnexectomy, a median incision below the umbilicus is made, followed by a cesarean section. In our case, the surgical approach was laparotomy with oophorectomy because of the large size of the tumor and the early stage of pregnancy.

Due to the rarity of struma ovarii during pregnancy and the absence of reliable prognostic factors, there is currently no consensus on the management of this condition after initial surgical diagnosis. A minimum follow-up period of 10 years is recommended.

## 9. Conclusions

In conclusion, while struma ovarii rarely affects the outcome of a normal pregnancy, it should be considered in the differential diagnosis of any persistent ovarian mass detected during pregnancy. Further evaluation is necessary to rule out malignancy as the pregnancy itself can stimulate thyroid carcinoma originating in struma ovarii.

Struma ovarii presents with atypical characteristics, and there is a lack of comprehensive data in the published literature regarding its diagnosis, surgical management, adjuvant therapy, and follow-up evaluation.

Given the rarity and heterogeneity of these tumors, decisions regarding diagnosis and treatment should be individualized for each case. A multidisciplinary approach involving a team of specialists is essential. More studies are required to determine optimal diagnosis and treatment strategies for pregnancy-related struma ovarii.

## Figures and Tables

**Table 1:** Reported cases of pregnancy-related struma ovarii.

Reference	Year	No. Cases/ Study Type	Patient Age (Years)	Histopathological Diagnostic	Surgical Technique
Im et al. [11]	2023	Case report	32	PTC and SC in PSO	Left ovarian cystectomy
Ho, Nagaratnam [12]	2022	Case report	28	PTC in PSO	Laparoscopic right S-O with omentectomy and right pelvic LN sampling
Botros et al. [4]	2021	Case report	32	Thyroxine-producing PSO	Right S-O
Li et al. [5]	2021	Case report	39	SO and PS in pregnancy	N/A
Donato et al. [13]	2021	Case report	35	MSO with concurrent TC in pregnancy	Laparoscopic oophorectomy followed by total thyroidectomy
Schick, Van Antwerp [14]	2021	Case report	35	MSO with severe preeclampsia	Right cystectomy
Nagahara et al. [15]	2020	Case report	30	PSO	Laparoscopy-assisted ovarian cystectomy
Pepe et al. [10]	2019	Case report	19	PSO	Laparotomy with right adnexectomy
Feng et al. [16]	2019	1/48 cases/Retrospective study	N/A (21–41)	PSO	Laparoscopic tumor resection
Khalife et al. [17]	2019	Case report	31	SO complicated with ovarian torsion	Laparoscopic ROD of the twisted pedicle with a cystectomy
Lager et al. [18]	2018	Case report	30	Metastatic MSO during pregnancy	Tumor resection and thyroidectomy
Mascilini et al. [19]	2017	1/34 cases/Retrospective study	N/A	PSO	Surgically removed adnexal mass/NOS
Chandrasekar [20]	2016	Case report	27	SO complicating pregnancy	Left S-O and omental biopsy
Markowska et al. [21]	2015	Case report	N/A	PSO	Tumor enucleation
Merza et al. [22]	2015	Case report	28	Hyperthyroidism from PSO	Ovarian cystectomy
Oreopulu et al. [23]	2015	Case report	36	SO complicating pregnancy	Exploratory laparotomy with en bloc resection of the right adnexa
Bhanap, Kulkarni [24]	2014	Case report	24	PSO	Exploratory laparotomy with right S-O
Lee et al. [9]	2012	Case report	35	Metastatic FC in SO complicating pregnancy	Right ovarian cystectomy followed by total thyroidectomy and RAI therapy
Coughlin, Haddad [25]	2009	Case report	38	PSO as HG in pregnancy	Laparotomy with left oophorectomy
Netters et al. [26]	2008	Case report	41	PSO	Left adnexectomy
Dede et al. [27]	2007	1/68 cases/retrospective study	N/A	MSO in pregnancy	Cystectomy during cesarean section
Usta et al. [28]	2006	Case report	33	PSO with controlateral round ligament dermoid cyst	Ovarian cystectomy
Guven et al. [29]	2005	Case report	N/A	PSO	N/A
Sifakis et al. [30]	2003	Case report	31	SO complicating pregnancy	Surgically removed right ovarian mass/NOS
Mancuso et al. [31]	2001	Case report	31	PSO	Laparotomy with S-O
Moggian et al. [32]	1980	Case report	N/A	PSO	N/A

Abbreviations: PTC, papillary thyroid carcinoma; SC, stromal carcinoid; PSO, pregnancy-related struma ovarii; SO, struma ovarii; PS, peritoneal strumosis; MSO, malignant struma ovarii; TC, thyroid carcinoma; FC, follicular carcinoma; LN, lymph node; HG, hyperemesis gravidarum; S-O, salpingo-oophorectomy; ROD, right ovarian detorsion; NOS, no other specification; N/A, not available; RAI, radioactive iodine therapy.
